# Self-Rated Health: Inequalities and Potential Determinants

**DOI:** 10.3390/ijerph6092456

**Published:** 2009-09-15

**Authors:** Evangelos C. Alexopoulos, Mary Geitona

**Affiliations:** 1 Department of Public Health, Medical School, University of Patras, 26500 Rio Patras, Greece; E-Mail:ecalexop@upatras.gr; 2 Department of Economics, University of Thessaly, 38221 Volos, Greece

**Keywords:** self-rated health, health inequalities, social determinants, chronic diseases, Greece

## Abstract

Understanding social inequalities in health is of great importance; it provides the conceptual frame for investigating the social factors that affect health, together with empirical evidence for improving population health. Individual and socioeconomic data, disease related conditions and self rated health (SRH) ratings were collected from a representative sample of 1,000 participants in order to study health inequalities in Greece. 20.8% of men and 37.2% of women reported poor health status. Significant inequalities in SRH were observed. Strong associations of poor SRH with gender, age, insurance coverage and chronic diseases were identified. Social insurance scheme captured partly the effects of educational level, income and residence area in SRH in multivariate analysis. Respondents under chronic treatment and those suffering from cardiovascular, musculoskeletal and neurological/psychiatric disorders exhibited the highest risk of reporting poor SRH. Our findings provide decision-makers with insights into how to manage health inequalities by prioritizing preventive measures and consequently, progress towards the fair distribution of healthcare resources.

## 1. Introduction

Understanding and acting on social health inequalities has proven to be a difficult task. Despite the increase of average life expectancy and the continuing improvements in overall population health, research on social factors etiology and on the course of physical health seems to be an endless process, accompanied by a persistence of social health inequalities [1–3]. Under the increasingly broad array of psychosocial and environmental risk factors for health, it is recognized that understanding further the social and economic forces which shape individual socioeconomic position will provide additional insight and tools for social action. Within this framework, medical care and insurance, social relationships, roles and activities are considered major explanatory variables on the causal pathway between socioeconomic status and various health outcomes. Other factors are related to the processes of measurement of ill-health, to socio-economic conditions and material well-being, social class structure and selection, lifestyle behavior and to biomedical, cultural, psychosocial, psychological and environmental explanations [1–4]. It has been argued that, although materialist and behavioural explanations cannot be separated, abandoning the effort to make any distinction between the two would not help in the etiology for inequalities in health [4].

Self-rated health (SRH) has also received growing attention in the international literature. It focuses on the evaluation of a population’s health status or health related quality of life and an individual’s well-being. Numerous studies report that SRH is not only a valid and reliable measure of a population’s general health and well being but also a strong predictor of morbidity, mortality and health services utilization [5–13]. Despite the fact that the use of SRH as a single index of self-reported health measure is influenced by different reference levels and criteria of the respondents against which health is judged, it is generally considered to be a valuable source of data on a population health status [14]. Several socioeconomic, demographic, behavioral and psychosocial determinants have been found to be associated with SRH [13–20]. In addition, physical health and chronic diseases have been considered to be the main determinants that affect self-reported health [1,10,11]. Similarly, great inequalities in self-reported health at the individual or population level have been widely reported in many studies [2,9,10,13–19].

In Greece, despite the universal and compulsory coverage of the population, several studies have reported significant health inequalities in the access to and financing of healthcare [21–23]. Primary health care is provided by the Social Insurance Funds’ polyclinics and the National Health System (NHS) Health Centres in rural and semi-urban areas and by the public hospitals’ outpatient units. Hospital care is mostly provided by the NHS hospitals and is financed by the social insurance funds. IKA is the largest social insurance fund, covering more than 60% of the insured population working in the private sector and the only provider of primary health care in its own facilities. All other funds cover different professional groups and provide health care through the NHS facilities and contracted physicians from the private health sector. OGA is the social fund covering the agricultural population, OPAD covers the employees in the public sector, OAEE the self-employed and the remaining funds cover employees working in semi-public organisations, i.e., banks, electricity, telecommunication, transportation. The fragmentation in the provision of primary healthcare, the lack of comprehensiveness in the social insurance coverage, the regional inequalities in resource allocation and in the access to healthcare provision have resulted in additional out of pocket and informal payments in order to ensure easier access to and better health care. As a result, Greece is among the Organization for Economic Cooperation and Development (OECD) countries with the highest private spending, corresponding to 48% of total health expenditures [24].

A few studies have explored the relationship of SRH with health services utilization and patients’ reported outcomes in Greece [23,25]. However, no previous studies have addressed the association of SRH with disease related conditions and self-reported medication use, factors that have been found to be strong predictors of mortality, morbidity and chronic disease [18,26]. The aim of the present study was to explore the associations of a series of potential socio-demographic and disease related determinants with SRH and to identify socioeconomic health inequalities.

## 2. Study Population and Methods

This paper is based on the data of a cross-sectional household survey conducted in Greece between November 2004 and February 2005. Interviews were conducted across the whole of Greece in a representative sample of 1,000 individuals aged 17 years and over, using a multi-stage random sampling technique. Sample selection was based on the 2001 Census of the National Statistical Service of Greece stratified by age, sex and geographical location at place of residence. From a sampling frame of all telephone numbers listed in an electronic directory (Hellenic Telecommunications Organization), a systematic national sample of 1,000 participants (telephone numbers) stratified by geographical region was drawn, in order to allocate the number of interviews in each area according to its population size. Once selected, a telephone number was considered eligible if it was a residential connection. In Greece, 95% of households have at least one fixed telephone line. Ineligible telephone numbers such as business telephones were replaced by the subsequent residential entry while mobile phone numbers were excluded. Sampling continued until we had 1,000 completed interviews. The interviewers were instructed to follow standard procedures for replacing persons who were originally selected for the interview but were unavailable (e.g., incorrect phone number, not answering the phone, not at home, unwilling to participate in the survey). The person who initially answered the telephone was eligible to be interviewed if s/he was older than 17 years of age. To avoid over sampling of specific age or gender subgroups, whenever possible an attempt was made to sample a subject different by age and gender in each subsequent interview within the family telephone line. However, this was limited to less than 10% of the cases. Methodology used regarding the sample selection and data collection has also been published elsewhere [27].

The survey examined SRH and a series of potential determinants including medical conditions (disorders, medication use), and demographic and socioeconomic factors such as age, gender, marital and working status, education, area of residence, insurance coverage and household income. The categorization of patients with chronic or temporary disease was made according to the patients’ answers to the following questions: *Do you currently suffer from a disease? If yes, have you received medication for this disease on a continuous-permanent or a temporary-short term basis? Have you suffered from a disease in the past three months? If yes, have you received medication? Have you suffered from any health related condition/problem (e.g., high levels of LDL cholesterol) now or during the last three months? If yes, have you received any medication?* In addition, respondents who suffered from a disease were asked to report, in simple words, their disease/disorder and whether it was diagnosed by a physician. The disease status categories were mutually exclusive. Whenever an individual gave more than one response, we assigned him to the most severe disease status according to his perception. Perceived health was rated on a 5-point scale: very good, good, moderate, poor and very poor and the question asked was: *How do you rate your general state of health?* The five category dependent variables were grouped into two categories. The “good SRH” and the “poor SRH” categories, whereas moderate SRH was grouped with the poor health. Medical disorders were classified as chronic when requiring continuous therapy-medication use, or short-term when requiring therapy for a definite amount of time. Queries regarding the socioeconomic characteristics were based on a set of items drawn from the WHO health survey [25].

Net income was measured after deductions of taxes and social security premiums. Respondents’ educational level was classified into three categories according to their completed educational level. The first category corresponds broadly to the uneducated and the elementary educational level (primary education), the second to the lower and higher secondary education and the third category to the tertiary lower technical and higher education (college and university).

In the statistical analysis differences between categorical variables were tested with the chi-square test (*X*^2^). Logistic regression analysis was performed to evaluate the influence of determinants on SRH. Odds ratios (OR) with 95% confidence intervals were calculated as measure of association, adjusted for age and gender. For the initial selection of potential determinants for SRH bivariate logistic regression analysis was used with a significance level of p < 0.10. Subsequently, all independent variables that showed significant associations were included in the multivariate logistic regression model and retained when significant at p < 0.05 (Wald test). In the results, the final multivariate model is presented, as well as the OR for other variables when included separately in this multivariate model. An OR above one indicates that the likelihood of less than good (poor) SRH is higher with the presence of the specified determinant. Data analyses were conducted by means of the SPSS for Windows 16.1.0 statistical package.

## 3. Results

The total study population consisted of 486 males and 514 females. The representativeness of the sample was checked against demographic indicators (e.g., age, gender, area of residence) collected from the National Statistical Service of Greece (2001 Population Census). In our sample, males comprised 48.6%, compared to the 48.9% of males in the general population. Also, 41.5% were aged between 18 and 39 years old and 32.5% between 40 and 59 years old, compared to 41.1% and 31% in the general population, respectively. The geographical (according to the place of residence) distribution of the sample was similar to that of the Greek population.

The survey response rate was 55%, since some of the individuals considered eligible for the interview were unwilling to participate. In addition, approximately 4.5% of the telephone numbers were replaced as being business or non residential telephones. To avoid over sampling, a replacement of around 95 individuals (75% due to age and the rest due to gender over-sampling) was identified as necessary. In general, missing data is estimated at 7% or less, apart from the case of questions regarding income-related variable where missing data was higher.

Table 1 shows the general characteristics of the 1,000 respondents. Approximately 39% of the study subjects reported a monthly household income of less than €1,500 (euros); however, 40% of the study subjects did not answer this question. Almost all subjects were entitled to compulsory health insurance coverage according to their employment status, while less than 2% reported no insurance coverage. The latter demonstrated the participation of uninsured immigrants in our sample. Also, 16% of the subjects had an additional private insurance.

Table 2 shows the level and equality of perceived health status across different population characteristics. Poor perceived health was reported by 29% of the respondents. Significant differences among all variables under study were also reported in the SRH level. The elderly, females, those with lower educational level and income, widow/divorced and the insured by OGA reported significantly worse health status. Regarding the respondents’ treatment status during the three months preceding the survey, 286 (28.6%) subjects were under chronic and 83 (8.3%) under short-term prescribed medication use. In addition, 528 subjects reported the occasional self use of over the counter drugs (OTC) for minor symptoms, while 103 had not taken any medication. As expected, the prevalence of poor health status was greater in the respondents suffering from a disease and/or those under chronic treatment.

In the multivariate regression analysis, two models were considered (Table 3). The first one included treatment status and the second the specific disease category. In both models gender, age and health insurance were important factors associated with perceived health status. Specifically, females and elders had an increased risk of reporting poor health. Those insured in IKA and especially in OGA had an increased possibility to report poor health compared to those insured by other social funds. As expected, those under chronic treatment have a higher possibility (OR 7.16; 95% CI: 3.68 to 13.93) to report poor health, while various patterns of health ratings corresponded to different disease entities. Respiratory, genitourinary and endocrine disorders showed OR between 4 to 4.5, gastrointestinal and metabolic, neurological/psychiatric and musculoskeletal disorders between 5.2 and 5.4 and cardiovascular/cerebrovascular disorders varied between 5.9 and 15.1.

## 4. Discussion

This survey investigates the association of SRH with various determinants and aims to identify health-related socioeconomic inequalities in a representative sample of 1,000 Greek households. The results of the study provide evidence of the association of SRH with several demographic, socioeconomic and disease-related conditions. According to our findings, less than good (poor) SRH was reported by almost 1/3 of the respondents. Health ratings of the Greek population are in accordance with the Western Europe population profile which is between 20% and 55%, while in Eastern Europe and the former Soviet Union countries poor SRH lies around 70% [16,17].

Significant differences were observed in the level of SRH across all variables under study. Gender, age and health insurance seem to be important factors associated with perceived health status. Females and elders had an increased risk of reporting worst health. In addition, individuals insured in OGA had an increased possibility of reporting poor health. It seems that social insurance coverage captured partly the effects of educational level, income and residence area in SRH because those insured to OGA are farmers, less educated, live in rural areas and are less wealthy. As expected, those under chronic treatment have a higher risk of reporting worse health while significant variations in health rating are observed among the different disease entities.

Significant socioeconomic inequalities in SRH are reported among European countries, mostly associated with age, gender, educational level, occupation, income, social class and support, mortality, morbidity and medical conditions such as chronic diseases [4,10,13–17]. Health inequalities are also reported in a cross-national study in which Greece and other 21 European countries participated [19]. The results of this comparative study showed that education is associated with higher levels of SRH and emotional support is not significantly related to SRH without taking into consideration other socio-economic determinants.

Similar research, led by the WHO in Greece, examining SRH and the extent to which it is affected by socio-demographic factors, has found that older people, those with a lower income, with less education and women were more likely to rate their health as worse. The associations were however less strong for age, income and education compared to other European countries that participated in the same survey [25]. On the other hand, no associations were found with social insurance coverage. However, males and people with higher education level were over-represented in this study and no other factor was available for analysis in the study. Furthermore, the authors deduced the existence of major differences in the quality and quantity of primary healthcare services offered by the various health funds. This is consistent with our results and, given that the Greek population is universally insured for both primary and secondary health care, we did not separate the variable in our analysis.

According to our results, women and possibly widowed/divorced individuals are found to be at increased risk of poor SRH even though the latter did not reach statistical significance in multivariate analysis. Baltic countries’ men also declared better subjective health than women, with the only exception being found in Finland [16]. The same finding is also reported in Ukraine, adding also that, family relations are strongly associated with SRH [9]. Similar health inequalities were reported in Germany before the unification [17]. More specifically, marital status was associated with SRH in West German women, with those being separated, divorced or widowed being more likely to report poor SRH.

Income and education-related health inequalities have been widely observed with significant differentiations in the degree of their association with self-perceived health. Education has been found to be a strong determinant of various health outcomes in the former communist countries of Central and Eastern Europe, whereas for Western European countries income appears to be the strongest determinant [28–31]. These international studies’ findings are in agreement with our results based on two important considerations. First, Greece belongs to the EU countries and has the same social and cultural characteristics with them –even though surrounded by former communist countries. Second, income, which was expected to be a strong determinant of the existing health inequalities, is mediated by the universal distribution of various social and health benefits through the mandatory social insurance coverage. A British study [14] showed a marked social gradient in SRH with the prevalence of poor health in men and women in both manual and non-manual labor social classes; in the first case for people under 50 and in the second over 70 years of age. The findings of the McFadden study, with a sample size of more than 22,000 participants, are in accordance with the inequalities observed in our study. More specifically, OGA and IKA insured population had an increased possibility of reporting poor health since it mainly consists of farmers, manual and unskilled workers.

In Greece, no previous studies have addressed the association of SRH with self-reported diseases and medication use. In the international literature, several studies have considered the effect of chronic diseases on SRH, mostly associated with elderly people, and report physical health as the main determinant of SRH [1,4,5,10,13,15,18,26,32]. Our results are more in agreement with two related studies in which gastrointestinal, cerebrovascular, neurological and musculoskeletal conditions were found to lead to poor SRH among patients with chronic diseases [10,33].

The identification of the relation of SRH with the above mentioned factors adds to a growing body of evidence of socioeconomic inequalities in Greece. SRH has been found in a Greek study [23] to be the most important determinant of health services utilization when compared to other socioeconomic factors. More specifically, poor SRH, older people and women showed increased health services utilisation, whereas income appears to affect significantly utilisation only for individuals with lower income. Similar studies have found that low income, poor health status and low educational level are factors associated with higher use of healthcare utilization while higher-income groups seem to have better access to the provision of health care [21,34].

Most of our results are consistent with those of the international literature, even though surveys are varied in the research hypothesis, the sample selection and size, as well as the methodology used. On the other hand, heterogeneous results in different countries have been produced, suggesting that differences appear to depend on cross-cultural factors and artefact explanations [1,4,9,13,17,29]. There are some limitations that merit consideration. The use of interview surveys to monitor inequalities in health poses several problems related to the sampling selection, the procedures used for collecting data, the reliance on reporting by individuals, health conditions taken into account etc. [1,4,7,15,35,36]. Also, the time frame and resources available for the survey limited the number of subjects contacted to the minimum number of participants required for the sample to be nationally representative. The fact that the study was cross-sectional and that it could not therefore be used for investigating the temporal mechanisms between diseases/treatment conditions and SRH, should be reported as a limitation, resulting in the possibility that the relationships described may not be entirely unidirectional [36]. However, SRH used in this study has been evaluated for both its validity and reliability, since it has already been used in several studies in Greece [25,27]; the same question and response scales measuring were used and they were addressed to the same cultural and linguistic population groups [4,11,37]. Furthermore, our study was not able to examine other factors that may contribute to the poor perceived health and which should not be overlooked such as lifestyle habits, environmental pollution, social behavior and others [2–4]. A final limitation is that health status and diseases/treatment conditions were self-reported and the validity of some of the questions may not be high. However, it should be kept in mind that self-reporting was the only way to acquire knowledge about the respondents’ physical conditions that affect their health status.

## 5. Conclusions

This survey investigates the association of self rated health (SRH) with various determinants and aims to identify health-related socioeconomic inequalities in a representative sample of 1,000 Greek households. The results of the study provide evidence of the association of SRH with several demographic, socioeconomic and disease-related conditions. According to our findings, less than good (poor) SRH was reported by almost 1/3 of the respondents. The high prevalence of poor SRH in the elderly, females, farmers, manual and unskilled workers, those with lower educational level (based on insurance related characteristics) as well as those suffering from chronic diseases constitutes an important finding upon which prevention concentrated policies, affecting specific groups’ poor SRH, can be introduced. The amelioration of those population groups’ health related quality of life and well-being could lead to the decrease of the observed socioeconomic inequalities in health. Also the results of the study would provide decision-makers with insights into how to manage health inequalities and device preventive measures in the health policy priority setting.

Our findings should constitute a starting point for further assessments regarding the extent to which inequalities in SRH change over time. It is believed that similar national or/and international overviews and comparisons, should contribute to the decrease of health inequalities, the prioritization of public health policies and consequently, the fair distribution of healthcare resources. A longitudinal study is needed to confirm our findings and define more factors associated with poor health and socioeconomic inequalities in health. The necessity of scientific evidence on public attitudes towards health issues should be encouraged and be taken into account primarily for the creation of preventive measures against specific chronic conditions.

## Figures and Tables

**Table 1.** Individual characteristics of respondents (n = 1000).

**Respondent’s characteristics**	**Males** **(n = 486)** **n (%)**	**Females** **(n = 514)** **n (%)**

Age group		

18–39	208 (42.8)	207 (40.2)
40–59	161 (33.1)	164 (31.9)
60+	117 (24.1)	143 (27.8)

Educational level *		

College or University	140 (29.3)	99 (19.5)
Secondary education	197 (41.2)	208 (41.0)
Primary or none education	141 (29.5)	200 (39.4)

Family status		

Living alone	54 (11.2)	74 (14.6)
Living with others	429 (88.8)	438 (85.4)

Total household income (monthly)*		

<750 Euros	70 (14.4)	112 (21.8)
750–1,500 Euros	93 (19.1)	114 (22.2)
1,500+ Euros	116 (23.9)	90 (17.5)
Not answered	207 (42.6)	198 (38.5)

Residence area *		

Urban	315 (64.8)	262 (51.0)
Semi urban	52 (10.7)	81 (15.8)
Rural	119 (24.5)	171 (33.3)

Health Insurance Organizations **		

“IKA”	216 (44.4)	201 (39.1)
“OGA”	74 (15.2)	143 (27.8)
“OPAD”	71 (14.6)	67 (13.0)
“OAEE”	61 (12.6)	51 (9.9)
Other Funds	50 (10.3)	42 (8.2)
Private insurance (mostly as extra)	92 (18.9%)	65 (12.6)
No insurance coverage	11 (2.3)	8 (1.6)

* Comparisons between males and females (X^2^, p value < 0.05);

** IKA: private sector; OGA: agricultural population; OPAD: public sector; OAEE: self-employed.

**Table 2.** Level and equality of perceived health status across different population characteristics (n = 1000).

**Respondent’s characteristics**	**Good self-rated health** **(n = 707)** **%**	**P**

Gender		<0.001

Males	79.2	
Females	62.8	

Age group		<0.001

18–39	84.1	
40–59	71.6	
60+	48.5	

Educational level		<0.001

College or University	82.4	
Secondary education	76	
Primary or none education	56	

Family status I		0.011

Living alone	61.2	
Living with others	72.2	

Family status II		<0.001

Married	68.3	
Single	84.6	
Widow/divorced	47.5	

Net household income (monthly)		<0.001

<750 Euros	52.2	
750–1500	73.4	
1500+	79.6	
Not answered	73.3	

Residence area		<0.001

Urban	75.2	
Semi urban	70.7	
Rural	62.1	

Health Insurance Organisations		<0.001

“IKA”	70.9	
“OGA”	55.3	
“OPAD”	79	
“OAEE”	82.1	
Other Funds	79.3	

Additional Private Insurance		<0.001

Yes	82.2	
No	68.6	

Treatment status		<0.001

Chronic	38.1	
Temporary	77.1	
No medication	87.4	
Occasional use	84.3	

Disease status *		<0.001

Healthy	88.4	
Infectious diseases	76	
Respiratory	57.1	
Genitourinary	55	
Endocrine diseases	52.6	
Gastrointestinal and metabolic disorders	51.2	
Musculoskeletal	50	
neurological/psychiatric	44	
Hypertension/cerebrovascular	43.9	
Cardiovascular	24.5	

* Main subcategories included (n ≥ 20).

**Table 3.** Logistic Regression of Poor Health on Selected Determinants of Health.

**Poor self-rated health**	**Unadjusted** **OR (95%CI)**	**Adjusted for final model I^** **OR (95%CI)**	**Adjusted for final model II^** **OR (95%CI)**

Gender			

Males	1	1*	1*
Females	2.26 (1.70 to 3.00)	1.76 (1.27 to 2.43)	1.69 (1.20 to 2.38)

Age group			

18–39	1	1*	1*
40–59	2.10 (1.47 to 3.00)	1.54 (1.03 to 2.28)	1.59 (1.04 to 2.42)
60+	5.62 (3.93 to 8.05)	2.00 (1.28 to 3.12)	2.24 (1.40 to 3.60)

Health Insurance Organizations			

“IKA”	1.62 (1.16 to 2.25)	1.71 (1.17 to 2.48)	1.53 (1.04 to 2.26)
“OGA”	3.19 (2.20 to 4.62)	2.17 (1.42 to 3.33)	1.91 (1.24 to 2.97)
All others	1	1*	1*

Treatment status			

No medication	1	1*	
Occasional use	1.29 (0.69 to 2.42)	1.20 (0.63 to 2.27)	
Short term	2.05 (0.95 to 4.45)	1.83 (0.82 to 4.05)	
Chronic	11.23 (6 to 21.05)	7.16 (3.68 to 13.93)	

Disease status			

Healthy	1		1*
Infectious diseases	2.42 (0.93 to 6.28)		3.08 (1.15 to 8.23)
Respiratory	5.74 (2.33 to 14.16)		3.99 (1.54 to 10.05)
Genitourinary	5.57 (2.16 to 14.36)		4.17 (1.59 to 10.94)
Endocrine diseases	6.89 (3.85 to 12.33)		4.37 (2.35 to 8.09)
Gastrointestinal and metabolic disorders	7.31 (3.81 to 14.03)		5.20 (2.64 to 10.25)
Neurological/psychiatric	9.74 (4.24 to 13.25)		5.20 (2.16 to 12.55)
Musculoskeletal	7.66 (4.43 to 13.25)		5.40 (3.02 to 9.65)
Hypertension/cerebrovascular	9.78 (5.89 to 16.26)		5.88 (3.35 to 10.31)
Cardiovascular	22.97 (11.64 to 45.32)		15.08 (7.26 to 31.33)

Educational level			

College or University	1	1	1
Secondary education	1.47 (0.98 to 2.20)	1.22 (0.78 to 1.92)	1.31 (0.82 to 2.11)
Primary or none	3.67 (2.47 to 5.45)	1.45 (0.87 to 2.39)	1.62 (0.96 to 2.74)

Family status I			

Living alone	1	1	1
Living with others	0.61 (0.42 to 0.90)	0.89 (0.56 to 1.42)	0.96 (0.59 to 1.54)

Family status II			

Married	1	1	1
Single	0.39 (0.27 to 0.57)	0.96 (0.60 to 1.54)	1.01 (0.62 to 1.65)
Widow/divorced	2.39 (1.55 to 3.66)	1.37 (0.82 to 2.30)	1.24 (0.73 to 2.11)

Household income (monthly)			

1500+	1	1	1
750–1500	1.43 (0.95 to 2.13)	1.10 (0.69 to 1.74)	1.08 (0.68 to 1.74)
<750 Euros	3.58 (2.29 to 5.59)	1.19 (0.68 to 2.10)	1.34 (0.75 to 2.38)
Not answered	1.41 (0.89 to 2.23)	1.01 (0.60 to 1.71)	0.90 (0.53 to 1.54)

Private insurance			

Yes	1	1	1
No	2.05 (1.34 to 3.15)	1.40 (0.86 to 2.29)	1.35 (0.81 to 2.25)

Residence area			

Urban	1	1	1
Semi urban	1.26 (0.83 to 1.91)	0.73 (0.44 to 1.19)	0.75 (0.45 to 1.24)
Rural	1.85 (1.37 to 2.51)	1.05 (0.69 to 1.62)	1.09 (0.70 to 1.71)

*Model characteristics*			
*R Square (Nagelkerke)*		*0.337*	*0.337*
*Goodness of fit Test Sig. (Hosmer and Lemeshow)*		*0.730*	*0.871*

* covariates of the final model (p < 0.05)

Final model I: Gender, Age group, Health Insurance Organizations, Treatment status

Final model II: Gender, Age group, Health Insurance Organizations, Disease status

^ all other variables are adjusted for the final models I and II.
